# Association between physiotherapist sleep duration and working environment during the coronavirus disease 2019 pandemic in Japan: A secondary retrospective analysis study

**DOI:** 10.1371/journal.pone.0306822

**Published:** 2024-07-09

**Authors:** Fumito Morisawa, Yuji Nishizaki, Shuko Nojiri, Hiroyuki Daida, Tohru Minamino, Tetsuya Takahashi

**Affiliations:** 1 Department of Cardiovascular Biology and Medicine, Juntendo University Graduate School of Medicine, Tokyo, Japan; 2 Medical Japan, Pfizer Japan Inc., Tokyo, Japan; 3 Division of Medical Education, Juntendo University School of Medicine, Tokyo, Japan; 4 Medical Technology Innovation Center, Juntendo University, Tokyo, Japan; 5 Clinical Research and Trial Center, Juntendo University, Tokyo, Japan; 6 Faculty of Health Science, Juntendo University, Tokyo, Japan; 7 Department of Physical Therapy, Faculty of Health Science, Juntendo University, Tokyo, Japan; University of Pretoria, SOUTH AFRICA

## Abstract

Studies have reported that health care professionals experienced a lack of sleep during the coronavirus disease 2019 (COVID-19) pandemic and that such lack of sleep and working environment affect their performance. However, to the authors’ knowledge, no study has yet investigated the relationship between sleep duration and working environment among Japanese physiotherapists during the COVID-19 pandemic. This study retrospectively investigated the sleep duration of physiotherapists directly providing physiotherapy to patients with COVID-19 within the red zone and analyzed the association between sleep duration and working environment using logistic regression analysis. Among the 565 physiotherapists studied, the average sleep duration was 6 (6–7) h, and 381 (67.4%) had an average sleep duration of ≤6 h. Less experienced physiotherapists were 1.03 times more likely to sleep ≤6 h, and those in charge of patients with COVID-19 as the supervisor ordered were 0.64 times more likely to sleep ≤6 h. Moreover, physiotherapists with a significant increase in the frequency of internal online meetings and those who had been providing physiotherapy to patients with COVID-19 for >6 months were 2.34 and 2.05 times more likely to sleep ≤6 h, respectively. During the COVID-19 pandemic in Japan, two-thirds of the physiotherapists directly providing physiotherapy to patients with COVID-19 slept less than the recommended duration. This study highlights the need for appropriate workload and work hour management for physiotherapists according to their experience and workload, as well as establishing a medical care system that includes work rotation to ensure that the recommended sleep duration is satisfied.

## Introduction

In recent years, Japan has seen a need to create and improve the working environment for health care professionals (HCPs) [1]. In its guidelines for reviewing working hours, the Ministry of Health, Labour, and Welfare recommends reducing overtime and holiday work and introducing work interval to ensure sufficient time for living and sleeping [2]. In April 2024, the work style reform laws will apply a cap on overtime work for physicians [3, 4]. In line with this, guidelines have been issued to evaluate efforts to reduce working hours, including the establishment of appropriate labor management systems, thereby promoting reforms in work styles [5]. In fact, the guidelines for developing the work hour reduction plan for physicians [5] and working strategies proposed by the Japan Nursing Association to allow nurses to continue working [6] have emphasized the need to secure intervals between shifts. Moreover, research has shown that it is essential for HCPs to manage their working hours and secure enough time for life and sleep.

During the coronavirus disease 2019 (COVID-19) pandemic, insomnia [7] and sleep disorders [8] among HCPs had been reported, with 59.5% of HCPs experiencing lack of sleep [9]. Furthermore, evidence suggests that the poor working environment and lack of sleep had an impact on their work performance [10]. Aside from being a predictor of stress symptoms [11], depression [11], and major depressive disorder and its severity [12], sleep duration has also been associated with coronary artery disease [13–15]. Thus, the reported association between shorter total sleep duration and depressive symptoms and anxiety [7], as well as the correlation between sleep duration and wellness and health [16], should drive leaders to create work schedules that ensure adequate sleep duration [17].

Although guidelines recommend ≥7 h of sleep for adults and the elderly [18–20], the association between >9 h of sleep and health risks is uncertain for people without sleep deprivation or illness [21], and some studies reported that ≥9 h of sleep is associated with the risk of metabolic syndrome [22] and cardiovascular disease mortality [23, 24]. HCPs had an average sleep duration of only 6 h during the COVID-19 pandemic [25, 26], which was shorter than that before the COVID-19 pandemic [16]. While studies have suggested an association between burnout and decreased sleep duration among HCPs during the COVID-19 pandemic [25, 26], the association between sleep duration among HCPs and working environment during the COVID-19 pandemic has not been thoroughly investigated.

In our previous study, we found that physiotherapists had an average sleep duration of 6 h during the COVID-19 pandemic [27], which was similar to the that for other HCPs [25, 26], and that some physiotherapists had experienced decreased sleep duration and increased overtime hours. We hypothesized that working environment would be associated with sleep duration in physiotherapists as they provide physiotherapy and conduct daily work in an environment they had never experienced before the COVID-19 pandemic. The current study aimed to retrospectively investigate the association between sleep duration and working environment among physiotherapists directly providing physiotherapy to patients with COVID-19 within the red zone.

## Material and methods

### Study design

This study retrospectively analyzed the association between sleep duration and working environment among physiotherapists during the COVID-19 pandemic in Japan using web-based survey data from our previous study obtained between March 5, 2021 and March 29, 2021 [27]. The web-based survey targeted physiotherapists providing direct physiotherapy to patients with COVID-19 within the red zone at 487 medical facilities that provide rehabilitation medicine, including intensivist training facility of the Japanese Society of Intensive Care Medicine (191 medical facilities), special functioning hospitals (77 medical facilities), and regional medical care support hospitals (219 medical facilities). The special functioning hospital was approved by the Minister of Health, Labour and Welfare to provide advanced medical care, whereas the regional medical support hospital was approved by prefectural governors to provide emergency medical and other medical care to patients referred by other medical institutions [28]. We have sent the research cooperation request form and the research explanatory document by mail to the physiotherapy department directors of the 487 medical facilities, through whom we requested that all physiotherapists providing direct physiotherapy to patients with COVID-19 within the red zone participate in this voluntary web-based survey [27]. Physiotherapists who agreed to participate in the study after reading the research explanatory document consented to participate in the study by clicking the participation consent button on the first page of the web-based survey [27]. To examine the association between sleep duration and working environment among physiotherapists, physiotherapists were divided into two groups, namely those with an average sleep duration of ≤6 and ≥7 h, based on sleep duration recommendations [18–20]. We accessed anonymized data that can not identify individual participants on April 17, 2024, and this study was conducted in accordance with the Strengthening the Reporting of Observational Studies in Epidemiology statement.

### Survey item

The web-based survey items used were average sleep duration (h), physiotherapist characteristics, and factors related to the working environment. Items under physiotherapist characteristics included sex, age (years), physiotherapy experience (years), certification, and living together with their families. Items under working environment included average overtime hours per week, average vacations/holidays per month, physiotherapy situation for patients with COVID-19, circumstances of being in charge of patients with COVID-19, presence of an adviser regarding the COVID-19-related work or stress, changes in lifestyle compared to before the COVID-19 pandemic, desired support as a way to cope with stress, frequency of internal and external meetings, and requirements to further promote physiotherapy for patients with severe infectious diseases, such as those with COVID-19, in the future.

### Statistical analysis

The dependent variable was an average sleep duration of ≤6 h, whereas the independent variables were physiotherapist characteristics and working environment. The results are expressed as mean ± standard deviation or median (interquartile range: IQR) or as numbers and proportions (%). Baseline categorical and continuous variables were compared between the two groups (i.e., those with an average sleep duration of ≤6 and ≥7 h) using the chi-square test or Fisher’s exact test and the two-tailed Student’s t test or Wilcoxon rank sum test. The association between sleep duration and working environment was evaluated using univariate and multivariate logistic regression analyses. The variables we included in the multivariate analysis were those identified to be significant or potentially confounding factors in the univariate analysis. The frequency of internal and external online meetings was determined by selecting one of five options (significantly decreased, a little decreased, almost unchanged, a little increased, and significantly increased); however, the number of categories had to be reduced from 5 to 3 given the very small number of respondents for the “significantly decreased” and “a little decreased” categories during data analysis as described earlier. The analysis excluded outliers identified by scatter plots and physiotherapists who selected ≥9 h of sleep based on sleep duration recommendations [18–21] as well as previous studies regarding sleep duration and health risk [22–24]. All data were aggregated and analyzed using SAS version 9.4 (Statistical Analysis Software; SAS Institute, Cary, NC), with a p value <0.05 indicating statistical significance.

### Ethics declarations

Our previously conducted study [27] was reviewed and approved by the Research Ethics Committee of the Faculty of Health Sciences, Juntendo University (Approval number: 20–035) and was carried out in accordance with the ethical principles of the Declaration of Helsinki and Ethical Guidelines for Medical and Health Research Involving Human Subjects. The research explanatory document included details regarding data anonymization, voluntary participation, and publication of study results, and all participants reviewed this document prior to participation. Participants who agreed to participate in the study after reading the research explanatory document consented to participate in the study by clicking the participation consent button on the first page of the web-based survey. Only participants who provided consent approval were included in the study. The Research Ethics Committee of the Faculty of Health Sciences, Juntendo University, reviewed and approved this study as a secondary retrospective analysis conducted under an opt-out policy (Approval number: 24–001).

## Results

**Fig 1:**
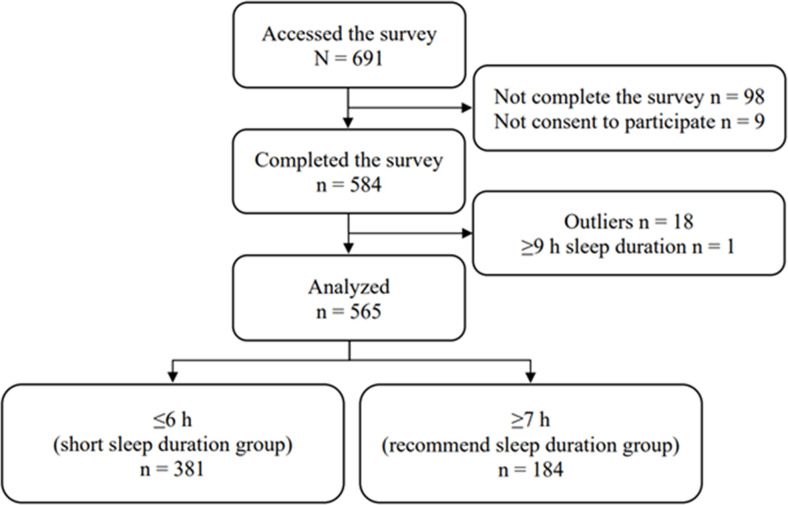
Study flowchart showing the participants and exclusions of physiotherapists.

This web-based survey was accessed by a total of 691 physiotherapists. 107 physiotherapists who did not complete the survey or did not consent to participate, 18 physiotherapists who were outliers, and 1 physiotherapist who reported sleeping for ≥9 h were excluded from analysis. Ultimately, 565 physiotherapists were included in the analysis (Fig 1).

Among the 565 physiotherapists (age, 38.2 ± 9.5 years; years of physiotherapy experience, 15.0 ± 9.1 years), 120 (21.2%) were female. The average sleep duration and average overtime hours per week were 6 (6–7) h and 3 (1–6) h, respectively. Regarding the survey items related to the situation of physiotherapy for patients with COVID-19, an average of 2 (1–3) patients with COVID-19 were managed daily, with an average of 40 (30–40) h of physiotherapy time per patient with COVID-19, and 83 (14.7%) physiotherapists had been providing physiotherapy to patients with COVID-19 for >6 months. Moreover, 237 (41.9%) physiotherapists were in charge of patients with COVID-19 by choice, whereas 220 (38.9%) physiotherapists by the supervisor’s order (Table 1).

**Table 1:** Comparison of physiotherapist characteristics according to sleep duration.

		Sleep duration			
	All	≤6 h (short sleep duration group)	≥7 h (recommend sleep duration group)		
	N = 565	n = 381 (67.4%)	n = 184 (32.6%)	p value	
Sex					
Male	445 (78.8%)	298 (78.2%)	147 (79.9%)	0.648	
Female	120 (21.2%)	83 (21.8%)	37 (20.1%)		
Age (years)	38.2 ± 9.5	39.4 ± 9.7	35.9 ± 8.8	<0.001	**
Physiotherapy experience (years)	15.0 ± 9.1	16.1 ± 9.2	12.6 ± 8.5	<0.001	**
Certification					
Certified physiotherapist	180 (31.9%)	124 (32.5%)	56 (30.4%)	0.614	
Professional physiotherapist†	47 (8.3%)	33 (8.7%)	14 (7.6%)	0.671	
Instructor of cardiac rehabilitation	120 (21.2%)	88 (23.1%)	32 (17.4%)	0.120	
Certified respiratory therapist	282 (49.9%)	196 (51.4%)	86 (46.7%)	0.295	
Not applicable	187 (33.1%)	124 (32.6%)	63 (34.2%)	0.689	
Living together with their families					
Yes	452 (80.0%)	303 (79.5%)	149 (81.0%)	0.686	
No	113 (20.0%)	78 (20.5%)	35 (19.0%)		
Average sleep time (hours)	6 (6–7)	6 (5–6)	7 (7–7)	<0.001	**
Average overtime hours per week	3 (1–6)	3 (1–7)	2 (1–5)	0.025	*
Average vacations/holidays per month	8 (8–9)	8 (8–9)	8 (8–9)	0.058	
Physiotherapy situation for patients with COVID-19					
Currently	211 (37.4%)	153 (40.2%)	58 (31.5%)	0.172	
1 to 2 weeks ago	93 (16.5%)	59 (15.5%)	34 (18.5%)		
3 weeks to 1 month ago	153 (27.1%)	95 (24.9%)	58 (31.5%)		
2 to 3 months ago	70 (12.4%)	47 (12.3%)	23 (12.5%)		
4 to 6 months ago	20 (3.5%)	12 (3.2%)	8 (4.4%)		
>6 months ago	18 (3.2%)	15 (3.9%)	3 (1.6%)		
Physiotherapy for patients with COVID-19 (total days)					
Approximately 1 to 7 days	130 (23.0%)	81 (21.3%)	49 (26.6%)	0.057	
Approximately 2 or 3 weeks	124 (22.0%)	80 (21.0%)	44 (23.9%)		
Approximately 1 month	89 (15.8%)	62 (16.3%)	27 (14.7%)		
1 to 6 months	139 (24.6%)	91 (23.9%)	48 (26.1%)		
>6 months	83 (14.7%)	67 (17.6%)	16 (8.7%)		
Average number of patients in charge per day	11.1 ± 4.0	11.1 ± 4.1	11.1 ± 3.9	0.923	
Average number of patients with COVID-19 in charge per day	2 (1–2)	2 (1–4)	2 (1–4)	0.783	
Average physiotherapy time per patient with COVID-19 (minutes)	40 (30–40)	40 (30–40)	40 (20–40)	0.729	
Physiotherapy prescriptions days per week for patients with COVID-19	5 (4–5)	5 (4–5)	5 (4–5)	0.721	
Circumstances of being in charge of patients with COVID-19					
By choice	237 (41.9%)	173 (45.4%)	64 (34.8%)	<0.001	**
Order from my supervisors	220 (38.9%)	125 (32.8%)	95 (51.6%)		
Others	108 (19.1%)	83 (21.8%)	25 (13.6%)		
Presence of adviser regarding the COVID-19-related work or stress					
Yes	508 (89.9%)	338 (88.7%)	170 (92.4%)	0.174	
No	57 (10.1%)	43 (11.3%)	14 (7.6%)		
Changes in lifestyle (comparison before the COVID-19 pandemic)					
Eating habits					
Became unhealthy	59 (10.4%)	46 (12.1%)	13 (7.1%)	0.130	
No change	455 (80.5%)	304 (79.8%)	151 (82.1%)		
Became healthy	51 (9.0%)	31 (8.1%)	20 (10.9%)		
Amount of alcohol					
Decreased	89 (15.8%)	58 (15.2%)	31 (16.9%)	0.795	
No change	364 (64.4%)	249 (65.4%)	115 (62.5%)		
Increased	112 (19.8%)	74 (19.4%)	38 (20.7%)		
Relaxation time					
Decreased	194 (34.3%)	141 (37.0%)	53 (28.8%)	0.064	
No change	329 (58.2%)	209 (54.9%)	120 (65.2%)		
Increased	42 (7.4%)	31 (8.1%)	11 (6.0%)		
Clinical activities (comparison before the COVID-19 pandemic)					
Conference in the rehabilitation department					
Significantly increased	15 (2.7%)	6 (1.6%)	9 (4.9%)	0.120	
A little increased	58 (10.3%)	40 (10.5%)	18 (9.8%)		
Almost unchanged	255 (45.1%)	169 (44.4%)	86 (46.7%)		
A little decreased	137 (24.3%)	92 (24.2%)	45 (24.5%)		
Significantly decreased	100 (17.7%)	74 (19.4%)	26 (14.1%)		
Internal online meetings					
Significantly increased	125 (22.1%)	99 (26.0%)	26 (14.1%)	0.005	**
A little increased	177 (31.3%)	118 (31.0%)	59 (32.1%)		
Almost unchanged	256 (45.3%)	158 (41.5%)	98 (53.3%)		
A little decreased	0 (0.0%)	0 (0.0%)	0 (0.0%)		
Significantly decreased	7 (1.2%)	6 (1.6%)	1 (0.5%)		
External online meeting					
Significantly increased	216 (38.2%)	158 (41.5%)	58 (31.5%)	0.040	*
A little increased	203 (35.9%)	127 (33.3%)	76 (41.3%)		
Almost unchanged	139 (24.6%)	92 (24.2%)	47 (25.5%)		
A little decreased	2 (0.4%)	0 (0.0%)	2 (1.1%)		
Significantly decreased	5 (0.9)	4 (1.1%)	1 (0.5%)		

Values are presented as mean ± standard deviation or number (percentage) or median (interquartile range).

*: p < 0.05,

**: p < 0.01

^†^: Professional physiotherapist is a higher qualification of certified physiotherapist.

Among the analyzed physiotherapists, 381 (67.4%) and 184 (32.6%) had an average sleep duration of ≤6 and ≥7 h, respectively. Among male and female physiotherapists, 298 (67.0%) and 83 (69.2%) had a sleep duration of ≤6 h, with no significant difference between them (p = 0.648). Table 1 summarizes the results of the comparison between those with a sleep duration of ≤6 and ≥7 h, whereas Table 2 shows the multivariate analysis. The ≤6-h group was significantly older (39.4 ± 9.7 vs. 35.9 ± 8.8; p < 0.001), had significantly more years of physiotherapist experience (16.1 ± 9.2 vs. 12.6 ± 8.5; p < 0.001) than the ≥7-h group, and sleep duration of ≤6 h was associated with years of physiotherapy experience (odds ratio [OR]: 1.03; 95% confidence interval [CI]: 1.01–1.06). The ≤6-h group demonstrated greater average overtime hours per week (3 [1–7] vs. 2 [1–5]; p = 0.025) than the ≥7-h group, but a sleep duration of ≤6 h was not associated with average overtime hours per week (OR: 1.03; 95% CI: 0.98–1.08). Furthermore, a significant difference in the circumstances of being in charge of patients with COVID-19 (p < 0.001) was observed between the two groups and sleep duration of ≤6 h was associated with being in charge of patients with COVID-19 as the supervisor ordered (OR: 0.64; 95% CI: 0.41–0.99). Lifestyle changes compared to before the COVID-19 pandemic indicated no significant differences in the proportion of physiotherapists who experienced changes in eating habits (p = 0.130), amount of alcohol (p = 0.795), or relaxation time (p = 0.064) were observed between the two groups. Clinical activities demonstrated a significant difference in the proportion of physiotherapists who experienced an increase or decrease in internal (p = 0.005) and external online meetings (p = 0.040) between the two groups, and sleep duration of ≤6 h was associated with a significant increase in the frequency of internal online meetings (OR: 2.34, 95% CI:1.32–4.13). However, no significant difference in rehabilitation department conferences were found between the two groups (p = 0.120). Moreover, a sleep duration of ≤6 h was associated with providing physiotherapy to patients with COVID-19 for >6 months (OR: 2.05; 95% CI: 1.04–4.06).

**Table 2:** Relationship between short sleep duration and working environment among physiotherapists using logistic regression analysis.

		Univariate	Multivariate
		OR (95% CI)	OR (95% CI)
Sex	Female	1.11 (0.72–1.71)	1.29 (0.81–2.06)
Physiotherapy experience (years)		1.05 (1.03–1.07)	1.03 (1.01–1.06)
Average overtime hours per week		1.06 (1.01–1.11)	1.03 (0.98–1.08)
Average vacations/holidays per month		0.86 (0.75–0.97)	0.91 (0.79–1.04)
Physiotherapy for patients with COVID-19 (total days)			
Approximately 1 to 7 days		1 (reference)	1 (reference)
Approximately 2 or 3 weeks		1.10 (0.66–1.83)	1.17 (0.69–2.00)
Approximately 1 month		1.39 (0.78–2.47)	1.35 (0.73–2.48)
1 to 6 months		1.15 (0.70–1.89)	1.14 (0.67–1.94)
>6 months		2.53 (1.32–4.86)	2.05 (1.04–4.06)
Circumstances of being in charge of patients with COVID-19			
By choice		1 (reference)	1 (reference)
Order from my supervisors		0.49 (0.33–0.72)	0.64 (0.41–0.99)
Others		1.23 (0.72–2.09)	1.23 (0.70–2.14)
Internal online meetings (comparison before the COVID-19 pandemic)			
Almost unchanged/a little decreased/significantly decreased		1 (reference)	1 (reference)
A little increased		1.21 (0.81–1.80)	1.43 (0.92–2.22)
Significantly increased		2.30 (1.40–3.79)	2.34 (1.32–4.13)
External online meeting (comparison before the COVID-19 pandemic)			
Almost unchanged/a little decreased/significantly decreased		1 (reference)	1 (reference)
A little increased		0.87 (0.56–1.36)	0.66 (0.41–1.08)
Significantly increased		1.42 (0.90–2.24)	0.79 (0.46–1.35)

OR: Odds Ratio, CI: Confidence Interval

## Discussion

In the current study, 67.4% of the physiotherapists had an average sleep duration of ≤6 h. Short sleep duration in HCPs has been significantly associated with long work hours and increased workload [29]. During the COVID-19 pandemic, working in a high-risk environment, such as providing direct front-line care to patients with COVID-19, had been identified as a risk factor associated with insomnia [30]. The current study, which included physiotherapists providing direct physiotherapy to patients with COVID-19, found that those who had an average sleep duration of ≤6 h had significantly more average overtime hours per week than did those with an average sleep duration of ≥7 h. Therefore, physiotherapists who averaged ≤6 h of sleep could have had a heavy workload and that their sleep duration was short due to increased overtime work and, among other reasons, non-work-related factors, including family commitments. Given the significant association between a short sleep duration and quality of patient care and patient safety [31], we believed it essential that each HCP obtain sufficient sleep. The consensus statement on sleep duration states that ≥7 h of sleep is necessary for optimal health promotion [21]. To ensure the health of each physiotherapist and the provision of safe and high-quality medical care to patients, ensuring sufficient sleep duration by adjusting work hours and workloads is imperative.

The current study found that years of physiotherapy experience was associated with sleep duration. During the COVID-19 pandemic, physiotherapists were required to implement thorough countermeasures for infection [32] and provide physiotherapy with personal protective equipment to patients with COVID-19 [33], which made normal procedures more time-consuming than usual and created an environment physiotherapists had never experienced before the COVID-19 pandemic. Furthermore, physiotherapists with skill are required to provide appropriate support and supervision to junior physiotherapists, and the involvement of senior physiotherapists are required when the appropriateness of physiotherapy for patients is identified [33]. One study revealed that only 25% of physicians include residents who are willing to participate in the COVID-19 duty [34]. HCPs in leadership positions were invigorated by seeing other HCPs who volunteered to work and communicate with colleagues and worked to make the lives of staff and patients safer with communicating with colleagues, they had difficulty turning off work [35]. In the current study, physiotherapists in charge of patients with COVID-19 by choice had an average physiotherapy experience of 17.1 years, whereas those in charge of patients with COVID-19 by order of their supervisors had an average physiotherapy experience of 11.0 years. Therefore, our findings suggest that mid-career physiotherapists with experience in physiotherapy at each medical institution took the initiative in dealing with COVID-19, including providing physiotherapy to patients with COVID-19, and worked under a tense, stressful, and difficult environment that required them to provide physiotherapy while implementing strict countermeasures for infection. The present study showed that older physiotherapists exhibited a shorter sleep duration, and sleep duration was associated with physiotherapists who provided >6 months of total physiotherapy to patients with COVID-19, suggesting that the high number of opportunities to provide physiotherapy directly to patients with COVID-19 during the COVID-19 pandemic may have affected sleep duration. We had previously reported that burnout among physiotherapists during the COVID-19 pandemic was associated with fewer years of physiotherapy experience, highlighting the need for support according to years of physiotherapy experience [27]. Therefore, aside from optimal support according to years of physiotherapy experience, appropriate support according to physiotherapist age should also be considered, which may include workload reduction among older HCPs in consideration of work environment and content. In line with this, a previous study had also found an association between short sleep duration and older age among HCPs [29].

The present study found that physiotherapists in charge of patients with COVID-19 by choice slept less. The COVID-19 pandemic had a considerable impact on the medical care delivery system in Japan [36] such that some HCPs had to leave their workplaces after becoming infected with COVID-19 or coming into close contact with patients with COVID-19 [37]. Under such circumstances, HCPs who could come to work were necessary to maintain the medical care delivery system. A certain number of physiotherapists voluntarily managed patients with COVID-19, not out of obligation due to the facilities being short-staffed, but out of passion and considering each medical facility’s situation. In the current study, physiotherapists in charge of patients with COVID-19 by choice averaged 4.75 h in overtime, whereas those in charge of patients with COVID-19 by order of their supervisor averaged 3.41 h in overtime. Therefore, physiotherapists in charge of patients with COVID-19 by order of their supervisor had their total workload adjusted in consideration of their COVID-19-related workload, whereas those in charge of patients with COVID-19 by choice were actively involved in their work and may have had a heavy workload. Considering the relationship between sleep and work strain [38], some physiotherapists in charge of patients with COVID-19 by choice may have worked under high tension and stress, which may have affected their sleep duration. As such, it is necessary to control the workload of physiotherapists in charge of patients with COVID-19.

The current study found that an increase in the frequency of internal online meetings was associated with sleep duration. Since the COVID-19 pandemic, internal, and external meetings have shifted rapidly from face-to-face to online. While online meetings have several advantages, such as the ability to join from anywhere without considering travel and move time in the hospital, which has led to a more efficient use of time, web meeting fatigue has been reported [39, 40]. During the COVID-19 pandemic, there was an increase in workload in the form of meetings on countermeasures for COVID-19 and infection prevention, including personal protective equipment wearing. Moreover, online meetings with care managers at other medical facilities regarding the post-discharge management of patients were held during work hours, whereas internal meetings were held online during non-work hours. One study showed the number of meetings was associated with fatigue and workload [41]. Apart from their usual work, the increased workload caused by the countermeasures for COVID-19 prevention and related meetings, as well as the holding of meetings during non-work hours, may have contributed to fatigue and workload, thereby promoting stress, and affecting sleep duration.

A Public Health Emergency of International Concern regarding COVID-19 was declared in January 2020 [42] and ended in May 2023 [43]. However, there continues to be a need for prevention, control, and management of the COVID-19 [44]. The guidance on the physiotherapy management of patients with COVID-19 recommends adjusting the staffing of physiotherapists according to the number of patients with COVID-19 [33], and further World Health Organization guidance requires rotation between high-stress and low-stress works [45]. Moreover, securing staff and hazard pay was among the top priorities for support measures for COVID-19-related HCPs [46]. In the current study, physiotherapists answered the survey items of desired support to cope with stress and requirements to further promote physiotherapy for patients with severe infectious diseases, and 71.7% of the physiotherapists desired a hazard pay as a means of coping with stress, whereas 71% required more workforce to promote further physiotherapy for patients with severe infectious diseases, such as those with COVID-19 (S1 Table). In the future, countermeasures need to be implemented according to infection status, and a medical care system that includes monitoring and management of daily work, including meetings and workload of each physiotherapist, and appropriate staffing including work rotations needs to be established.

This study has several limitations worth noting. First, this study did not evaluate sleep disorders among physiotherapists, such as insomnia, and poor sleep quality, using indices such as the Pittsburgh Sleep Quality Index [47]. Therefore, we could not evaluate the association of sleep disorders and sleep quality among physiotherapists with their working environment, as well as assess underlying sleep disorders or working during COVID-19 pandemic to cause the reported sleep duration. Second, this study did not investigate COVID-19 infections and co-morbidities in general, especially depression or other mental health problems that affect sleep quality and duration among physiotherapists, and we could not identify such infections and co-morbidities with their sleep duration. Finally, the results of this study cannot be generalized given that this was a voluntary survey of physiotherapists who provided direct physiotherapy to patients with COVID-19 within the red zone at 487 medical facilities in Japan.

## Conclusions

During the COVID-19 pandemic in Japan, two-thirds of the physiotherapists directly providing physiotherapy to patients with COVID-19 slept less than the recommended duration. Our study found that years of physiotherapist experience, being in charge of patients with COVID-19, and the increase in the number of online meetings were associated with sleep duration among physiotherapist. For any future pandemic that may occur and workload in general during the pandemic, these findings highlight the need for appropriate management of workload and work hours according to the experience and workload of each physiotherapist and for establishing a medical care system that includes work rotation to ensure that physiotherapists get the recommended sleep duration.

## Supporting information

S1 Table Comparison of desired support to cope with stress and requirements for promoting further physiotherapy in patients with severe infectious diseases such as those with COVID-19, in the future according to sleep duration of physiotherapist. (DOCX)
