# Supplementary material for: Association between physiotherapist sleep duration and working environment during the coronavirus disease 2019 pandemic in Japan: A secondary retrospective analysis study
Source: PLoS One. 2024 Jul 9;19(7):e0306822. doi: 10.1371/journal.pone.0306822 (PMC11233007; doi:10.1371/journal.pone.0306822)
Supplement: S1 Table — (DOCX) [file pone.0306822.s001.docx]

**Association between physiotherapist sleep duration and working environment during the coronavirus disease 2019 pandemic in Japan: A secondary retrospective analysis study**

Fumito Morisawa, Yuji Nishizaki, Shuko Nojiri, Hiroyuki Daida, Tohru Minamino, and Tetsuya Takahashi

**S1 Table. Comparison of desired support to cope with stress and requirements for promoting further physiotherapy in patients with severe infectious diseases such as those with COVID-19, in the future according to sleep duration of physiotherapist**

|  | |  |  | Sleep duration | | | | |
| --- | --- | --- | --- | --- | --- | --- | --- | --- |
|  | |  | All | ≤6 h  (short sleep duration group) | ≥7 h  (recommend sleep duration group) |  |  | |
|  | |  | N = 565 | n = 381  (67.4%) | n = 184  (32.6%) | p-value | | |
| Desired support to cope with stress | | |  |  |  |  | |  |
|  | Reduce overall workload | | 289 (51.2%) | 189 (49.6%) | 100 (54.4%) | 0.291 | |  |
|  | Increase staff | | 206 (36.5%) | 141 (37.0%) | 65 (35.3%) | 0.697 | |  |
|  | Gratitude and respect from others | | 143 (25.3%) | 91 (23.9%) | 52 (28.3%) | 0.262 | |  |
|  | Hazard pay | | 405 (71.7%) | 259 (68.0%) | 146 (79.4%) | 0.005 | | * |
|  | Proper education to prevent infection, increased educational opportunities, access to educational resources | | 197 (34.9%) | 131 (34.4%) | 66 (35.9%) | 0.728 | |  |
|  | Child care support | | 40 (7.1%) | 24 (6.3%) | 16 (8.7%) | 0.298 | |  |
|  | Counseling | | 56 (9.9%) | 42 (11.0%) | 14 (7.6%) | 0.203 | |  |
|  | Others | | 45 (8.0%) | 36 (9.5%) | 9 (4.9%) | 0.061 | |  |
| Requirements for promoting further physiotherapy in patients with severe infectious diseases, such as those with COVID-19, in the future | | |  |  |  |  | |  |
|  | Workforce | | 401 (71.0%) | 284 (74.5%) | 117 (63.6%) | 0.007 | | * |
|  | Understanding of physiatrist | | 140 (24.8%) | 102 (26.8%) | 38 (20.7%) | 0.114 | |  |
|  | Understanding of the rehabilitation profession manager | | 204 (36.1%) | 143 (37.5%) | 61 (33.2%) | 0.310 | |  |
|  | Hospital owners’ trust in the rehabilitation department | | 277 (49.0%) | 194 (50.9%) | 83 (45.1%) | 0.196 | |  |
|  | Awareness-raising activities by the Japanese Physical Therapy Association | | 173 (30.6%) | 122 (32.0%) | 51 (27.7%) | 0.298 | |  |
|  | Establishment of evidence for physiotherapy | | 408 (72.2%) | 269 (70.6%) | 139 (75.5%) | 0.219 | |  |
|  | Increase opportunities to learn about physiotherapy for critically ill patients, including those with infectious diseases | | 363 (64.3%) | 253 (66.4%) | 110 (59.8%) | 0.124 | |  |
|  | Physiotherapy qualification system for critically ill patients | | 130 (23.0%) | 87 (22.8%) | 43 (23.4%) | 0.887 | |  |
|  | Others | | 42 (7.4%) | 30 (7.9%) | 12 (6.5%) | 0.566 | |  |

Values are presented as number (percentage)

*: p < 0.01
